# Development and validation of ACTE-MTB: A tool to systematically assess the maturity of molecular tumor boards

**DOI:** 10.1371/journal.pone.0268477

**Published:** 2022-05-13

**Authors:** Tara M. Love, Daniel A. Anaya, Matthew S. Prime, Luke Ardolino, Okan Ekinci

**Affiliations:** 1 Roche Information Solutions, Roche Diagnostics Corporation, Santa Clara, California, United States of America; 2 Department of Gastrointestinal Oncology, Moffitt Cancer Center, Tampa, Florida, United States of America; 3 Roche Information Solutions, Roche Diagnostics Corporation, Basel, Switzerland; 4 Department of Medical Oncology, Garvan Institute of Medical Research, Darlinghurst, NSW, Australia; 5 St. Vincent’s Clinical School, University of New South Wales, Darlinghurst, NSW, Australia; 6 School of Medicine, University College Dublin, Dublin, Ireland; Qatar University College of Medicine, QATAR

## Abstract

Molecular tumor boards (MTBs) require specialized activities to leverage genomic data for therapeutic decision-making. Currently, there are no defined standards for implementing, executing, and tracking the impact of MTBs. This study describes the development and validation of ACTE-MTB, a tool to evaluate the maturity of an organization’s MTB to identify specific areas that would benefit from process improvements and standardization. The ACTE-MTB maturity assessment tool is composed of 3 elements: 1) The ACTE-MTB maturity model; 2) a 59-question survey on MTB processes and challenges; and 3) a 5-level MTB maturity scoring algorithm. This tool was developed to measure MTB maturity in the categories of Access, Consultation, Technology, and Evidence (ACTE) and was tested on 20 MTBs spanning the United States, Europe, and Asia-Pacific regions. Validity testing revealed that the average maturity score was 3.3 out of 5 (+/- 0.1; range 2.0–4.3) with MTBs in academic institutions showing significantly higher overall maturity levels than in non-academic institutions (3.7 +/- 0.2 vs. 3.1 +/- 0.2; P = .018). While maturity scores for academic institutions were higher for Consultation, Technology, and Evidence domains, the maturity score for the Access domain did not significantly differ between the two groups, highlighting a disconnect between MTB operations and the downstream impact on ability to access testing and/or therapies. To our knowledge, ACTE-MTB is the first tool of its kind to enable structured, maturity assessment of MTBs in a universally-applicable manner. In the process of establishing construct validity of this tool, opportunities for further investigation and improvements were identified that address the key functional areas of MTBs that would likely benefit from standardization and best practice recommendations. We believe a unified approach to assessment of MTB maturity will help to identify areas for improvement at both the organizational and system level.

## Introduction

As comprehensive genomic profiling and precision oncology become more routinely integrated into clinical practice, molecular tumor boards (MTBs) are playing an increasingly important role in clinical decision-making. MTBs provide a multidisciplinary forum for dedicated discussions on molecular data to guide diagnostic and therapeutic decisions tailored to a patient’s molecular profile. Recently, Kato et al demonstrated that adherence to MTB recommendations was associated with significantly higher rates of treatment matching which translated into improved overall survival [1]. However, in spite of the clearly established clinical utility of MTBs, there remains significant site-to-site variation in MTB-recommended treatment decisions [2]. In addition, there is currently no widely-accepted set of standards or best practices for implementing, coordinating, and measuring the effectiveness of an MTB.

To begin to address this need, we adapted the Capability Maturity Model Integration (CMMI) strategy to assess MTB processes. The CMMI approach is a model focused on measuring maturity levels of processes and behaviors in order to benchmark and empower organizations to adopt improved, streamlined actions and workflows, ensuring highest quality and lowest risk [3]. The CMMI maturity framework is composed of 5 different maturity levels that describe the state of one or more processes, from an initial, largely unstructured management state (Level 1) to a well-optimized management state (Level 5). Between levels 1 and 5 are intermediate states where management of processes essentially becomes less reactive and more proactive as maturity increases [3]. This CMMI-based 5-level maturity grading system can be applied at the level of a project, division, or entire organization, making it a versatile and powerful means of structuring processes, determining improvements, and tracking such improvements over time.

Maturity models predicated on the CMMI framework have shown success in identifying areas requiring process improvements in the implementation of multidisciplinary care in oncology practices [4–6]. MTBs specifically pose a unique set of challenges to the practice of precision medicine and involve highly specialized multidisciplinary teams (MDT). A maturity model recently published by Liu et al. was developed and validated for MDTs to perform self-assessments via survey to measure performance and track improvement in processes over time [6]. However, to date there is not a tool to perform similar maturity assessments for MTBs, resulting in a missed opportunity to identify areas of non-conformity with agreed processes and established standards. Our primary goals were therefore to develop a tool to specifically measure the maturity level of MTBs within an institution, as well as to identify specific domains of variation when considering implementation and dissemination of MTBs globally.

In this report, we describe the development of the ACTE-MTB maturity assessment tool and the subsequent validation of this tool to serve as a valuable asset to any healthcare organization or system seeking to perform an assessment of their MTB maturity. With five maturity levels in each of the categories of Access, Consultation, Technology, and Evidence, the ACTE-MTB tool was used to assess 20 MTBs spanning institutions in the United States, Europe, and Asia-Pacific regions. During this process, specific areas in each category were identified for further investigation and improvement.

## Materials and methods

This study was reviewed by the Western Institutional Review Board (IRB) and determined to be exempt under 45 CFR § 46.104(d)(2) (WCG IRB Work Order #1-1511111-1). All relevant data and statistical calculations underlying the results described in this study can be found in the S1 File. The ACTE-MTB maturity assessment tool was created in three phases consisting of tool development, initial validity testing on 6 MTBs, and further testing on an additional 14 MTBs (Table 1). MTBs for this study were recruited via a snowball sampling approach that originated within the Roche network of associated healthcare providers across Diagnostics and Pharma divisions and extended further among external health care provider networks. The aim was to have a representative balance of MTBs housed in academic vs. non-academic institutions spanning the United States, Europe, and Asia-Pacific. One of four institution types (academic medical center, community hospital, specialized cancer clinic, or private practice) was self-reported by each selected MTB. Academic medical centers comprised the academic category while community, specialized, and private institutions comprised the non-academic category.

**Table 1:** A 3-phased strategy for development and validity testing of the ACTE-MTB maturity model.

Phase	Input	Output	Established Validity
**1: Tool development**	• Expert interviews (n = 15)	• ACTE-MTB model: 4 domains (Access, Consultation, Technology, and Evidence)	Face^a^ Content^b^
	• Observations of MTBs (n = 8)	• 59-question survey	
	• Select literature review [7–11]	• 5-level maturity scoring algorithm	
**2: Initial testing**	Completed surveys for 6 MTBs	Domain-specific and overall maturity scores significantly differed for 6 different MTBs.	Construct^c^
**3: Further testing**	• Completed surveys for 14 additional MTBs for a total of 20 MTBs (9 academic; 11 non-academic^d^)	Result: Significantly higher overall maturity scores were observed for academic MTBs vs. non-academic MTBs.	Construct
	• Hypothesis: Academic MTBs have higher overall maturity scores than non-academic MTBs.		

^a^Face validity refers to the degree to which ACTE-MTB appears (at face-value) to be measuring MTB maturity.

^b^Content validity refers to the degree to which ACTE-MTB covers the relevant MTB activities and processes (content) to allow for measuring MTB maturity.

^c^Construct validity refers to the degree to which ACTE-MTB is truly measuring MTB maturity (the construct) as intended.

^d^Academic MTBs are defined as MTBs housed in academic medical centers while non-academic MTBs are defined as MTBs housed in community, specialized, and private institutions.

### Phase 1: Tool development

Phase 1 began by gathering insights on various MTB processes and challenges via expert interviews (n = 15), direct observations of MTBs (n = 8), and a review of select literature [7–11]. The 15 interviewed experts represented a mix of healthcare providers and scientists with extensive MTB experience who were either internal (n = 4) or external (n = 11) to Roche. External experts were compensated at an hourly rate consistent with fair market value rates for their specialty and country. From the gathered insights and learnings around MTB operations and workflows, 4 major themes foundational to all MTBs emerged: Access, Technology, Consultation, and Evidence. And from these themes, 3 outputs were conceived: 1) the ACTE-MTB maturity model; 2) a corresponding 59-question survey on MTB infrastructure and processes; and 3) a scoring algorithm that calculates maturity scores between levels 1 and 5. These components were then outlined and reviewed in follow-up sessions with 8 of the original 15 experts to support the establishment of both face and content validity. Face validity refers to the degree to which the ACTE-MTB tool appears (at face-value) to be measuring MTB maturity, while content validity refers to the degree to which ACTE-MTB covers the relevant MTB activities and processes (content) to allow for true measurement of MTB maturity. Each of the outputs are described in detail below.

#### ACTE-MTB maturity model development

**Fig 1:**
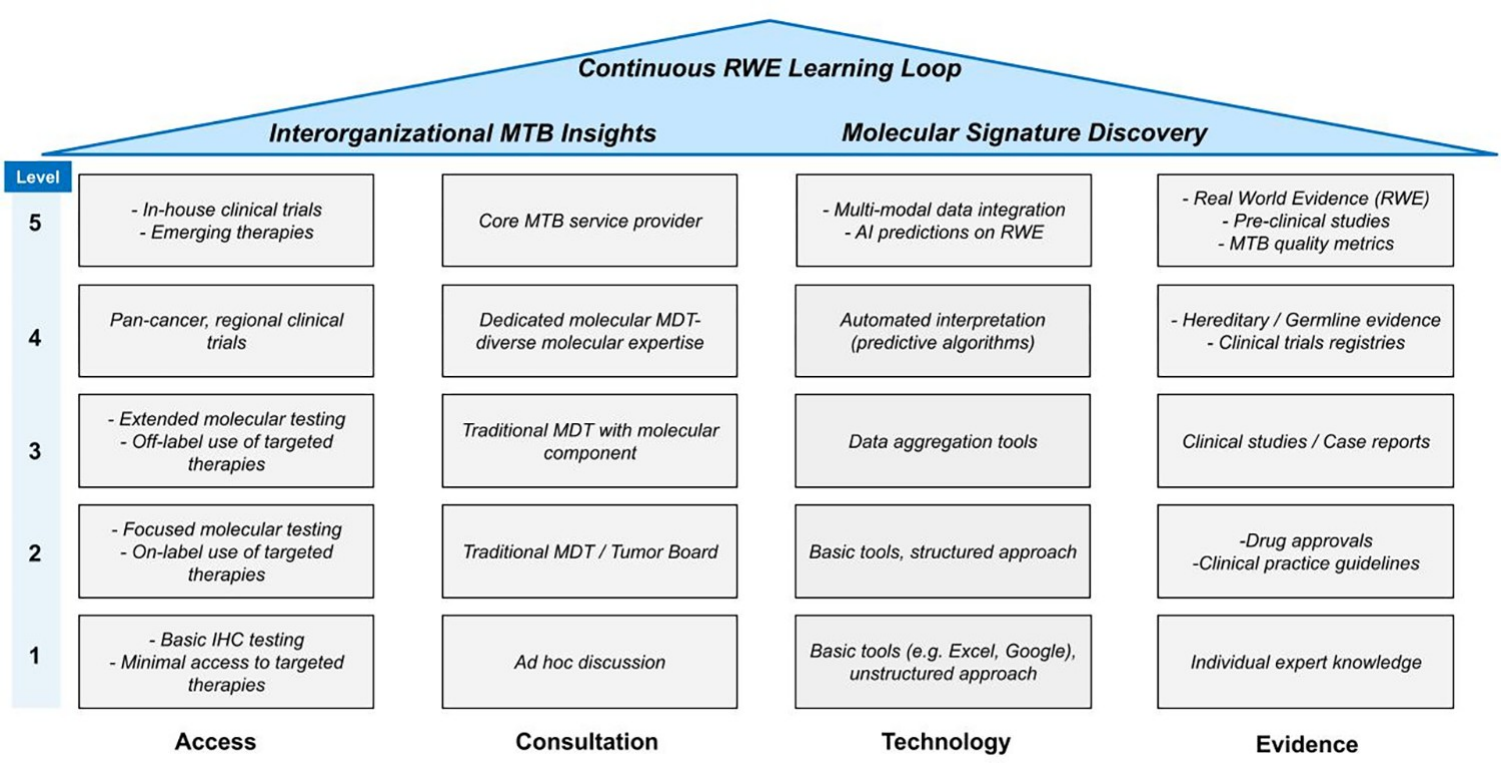
The ACTE-MTB maturity model. **MTB**: Molecular Tumor Board; **IHC**: Immunohistochemistry; **MDT**: Multi-Disciplinary Team; **RWE**: Real World Evidence.

The ACTE-MTB maturity model was created by starting with the well-validated 5-maturity-level CMMI framework [3] and a “house” representation published by Orenstein et al. to depict a maturity model for clinical decision support systems [12]. Key elements from these models were then adapted to the insights gathered on MTB infrastructure, processes, and challenges to create the ACTE-MTB model. ACTE-MTB was named to include the first letter of each of 4 domains that surfaced as being foundational to development of new MTBs, improved uptake of existing MTBs, and increased implementation of MTB recommendations into clinical practice by healthcare providers. These domains, Access, Consultation, Technology, and Evidence (ACTE), are represented as the four “pillars” of a house (Fig 1), where each pillar has 5 components or maturity levels with increasing complexity from bottom to top. By increasing maturity levels in each domain, it is believed that a system with advanced capabilities can eventually be achieved. Such capabilities comprise the “rooftop” of the model and may include interorganizational sharing of MTB insights, proactive discovery of new actionable molecular signatures, and continuous learning through the use of real-world evidence for molecularly guided clinical decision-making.

#### MTB survey development

A survey was created to include 59 possible questions in the following categories: Demographics, MTB Operations, MTB Patients, Molecular Testing, Molecular Alteration Interpretation, MTB Tools and Performance, and Optional Final Thoughts (S2 File). All questions in the required sections (n = 56) were structured into multiple-choice format, while the final 3 optional questions allowed for free-text responses. For subsequent testing phases (described below), this survey was administered between January and July 2021 as a self-reporting online survey to 20 participants identified via a snowball sampling of clinicians contributing to MTBs (Table 2). Each survey participant in the sampling consented to taking the survey by receiving and clicking on a link provided electronically by the requestor. In the survey instructions, participants were informed that their participation was strictly voluntary and that the data would remain anonymous, as names were not automatically collected and only general demographic details including country, institution type, and role(s) of respondents were captured. Participants were also encouraged to provide their individual experiences, views, and opinions vs. those of their institution. To ensure that individuals being surveyed were participants of MTBs, the Google Forms survey had a built-in exit mechanism for a “no” answer to the initial question of whether they participate in an MTB. For this study, all 20 respondents reported participation in an MTB with a survey completion rate of 100%. Data were collected automatically via the Google Forms interface, which, upon survey submission by each participant, auto-populated a spreadsheet with answers to the survey questions (S1 File).

**Table 2:** MTB demographics and participation in validity testing of the ACTE-MTB maturity model.

Country	Institution Type	Role(s) of Survey Respondent
***6 MTBs for Phase 2 testing of ACTE-MTB***		
Australia	Academic	Medical oncologist/Bioinformatician/CEO
China	Specialized center	Medical oncologist
Germany	Community	Medical oncologist
Germany	Private	MTB coordinator
United States	Academic	Medical oncologist
United States	Specialized center	Medical oncologist
		Organ specialist
***14 additional MTBs for Phase 3 testing of ACTE-MTB***		
Austria	Academic	Medical oncologist
China	Specialized center	Medical oncologist/Radiation oncologist/Organ specialist
China	Specialized center	Anatomic pathologist/Molecular pathologist/Clinical scientist
Taiwan	Academic	Medical oncologist
France	Academic	Clinical scientist
		MTB coordinator
France	Academic	Clinical scientist
		MTB coordinator
Germany	Community	Medical oncologist
Germany	Community	Medical oncologist
South Korea	Academic	Medical oncologist
United Kingdom	Specialized center	Medical oncologist
		Clinical scientist
United Kingdom	Community	Medical oncologist
United Kingdom	Academic	Anatomic pathologist
		Molecular pathologist
		Laboratory Specialist
United States	Academic	Pharmacist
United States	Private	Medical oncologist

**MTB**: Molecular Tumor Board; **CEO**: Chief Executive Officer

#### MTB maturity scoring algorithm development

The final output for Phase 1 was a scoring system for translation of responses to select survey questions into maturity scores between 1 and 5. Survey responses from a subset of 20 of the 59 questions were used to generate maturity scores. Five multiple-choice questions in each of the 4 ACTE-MTB domains, Access, Consultation, Technology, and Evidence (Table 3) were selected and validated through a process of review and feedback from 8 of the original 15 experts described above. Five points were allocated to each question and were distributed across a set of possible responses. Response types included increasing percentages, increasing quantities, increasing complexities, and binary answers (e.g., yes/no) and each set of responses for a given question was graded between 1 and 5 points (S1 File, S1 Table). The 5 levels in each domain of the ACTE-MTB model guided the grading of each response with 5 points corresponding to Level 5, 4 points to Level 4, and so forth.

**Table 3:** The 20 survey questions used for translation to maturity scores in the access, consultation, technology, and evidence domains.

**Access**				
What areas of clinical care are influenced by the MTB decisions?	Approximately what fraction of therapies recommended by the MTB are standard systemic therapies?	Approximately what fraction of therapies recommended by the MTB are off-label systemic therapies?	Of therapy recommendations made in the MTB, approximately what percentage are implemented?	In what way are MTB decisions enabling access and / or reimbursement for targeted therapies?
**Consultation**				
What percentage of patients have molecular / genomic information discussed as part of their case presentation?	Which roles typically participate in the MTB?	If your MTB has assigned functions, which role(s) perform the listed functions?	Is there a separate, dedicated "curation" meeting ahead of the MTB meeting where molecular alterations are vetted and prioritized?	What is the source of patients [internal vs. external] discussed at the MTB?
**Technology**				
Where [in-house vs. external] does molecular (genomic) testing occur?	What types of molecular tests are being discussed in the MTB?	What tools are used specifically for molecular data interpretation?	Which, if any MTB decisions and actions (recommendations, administered therapies, outcomes) are documented into a structured system such as an internal knowledge base, LIS/LIMS, or MDT software?	To what degree are MTB documented decisions accessible to participants [subset vs. all] post the meeting?
**Evidence**				
What inclusion criteria are used to assign a patient to the MTB?	What evidence (from molecular testing reports or otherwise) is used to prioritize molecular alterations for discussion in the MTB?	Do you participate in the initial interpretation of data derived from molecular (genomic) testing?	In what capacity is RWE from clinical practice being used or soon will be used to support MTB decisions?	What metrics (KPI) are used to track MTB use and impact?

**MTB**: Molecular Tumor Board; **LIS/LIMS**: Laboratory Information System / Laboratory Information Management System; **MDT**: Multi-Disciplinary Team; **RWE**: Real World Evidence; **KPI**: Key Performance Indicators

Each domain (with 5 questions per domain) had a maximum potential of 25 points, which were then evenly distributed among 5 maturity levels to result in maturity scores between 0 and 5.0 (S1 Fig). In rare cases where a single maturity survey question in a given category was not answered, the sum of existing points from 4 of the 5 questions was normalized over a denominator of 25. Overall maturity scores were calculated by taking the sum of points generated for each domain category and dividing by 4, thus representing an average across the four domains.

### Phase 2: Initial testing

In Phase 2, the 59-question MTB survey was administered to 6 clinicians representing 6 distinctive MTBs (Table 2) and the survey responses were scored for overall maturity and domain-specific maturity using the maturity scoring algorithm developed in Phase 1. The aim of this phase was to begin to establish construct validity i.e., the degree to which the ACTE-MTB assessment tool truly measures MTB maturity, as intended by the construct. For these first 6 MTBs, the goal was to simply establish that there was enough sensitivity in the scoring algorithm to obtain different maturity scores across different MTBs.

To visualize ACTE-MTB assessments and scores, radial stacked bar charts were created using modified code originally published by Observable (observablehq.com/, San Francisco, CA). In these plots, the 5 maturity levels were illustrated using a stoplight color scale, which was applied at the level of each of the 20 survey questions to illustrate progression from lower maturity (red) to higher maturity (green). A higher frequency of green observed in the radial plot therefore indicates higher maturity at the level of a single question, at the level of a specific domain, and overall. Using this color scale, deviations are easily spotted and highlight potential practical areas for follow-up discussions with survey participants.

### Phase 3: Further testing

In Phase 3, the ACTE-MTB assessment was further tested on 14 clinicians representing an additional 14 distinctive MTBs (Table 2) to further establish construct validity i.e., determine whether the ACTE-MTB tool could capably distinguish maturity level differences between overtly functionally-different MTBs. The MTBs were therefore stratified by institution type (academic vs. non-academic) and the hypothesis that maturity levels would be significantly higher for MTBs housed in academic institutions was tested. It was expected that maturity levels in academic institutions would be significantly higher given the greater likelihood of in-house testing, clinical trials, and research at these locations. Standard error or variance was calculated for maturity score averages and specified accordingly. For paired comparisons across 2 populations, the P-value was calculated using a 2-sample student’s t-test.

## Results

In Phase 1 of the development strategy for the ACTE-MTB assessment tool, insights were gathered from precision oncology expert opinions of MTBs, authors’ personal observations of MTBs, and a select literature review. These insights were then consolidated to create the ACTE-MTB maturity model, a 59-question scientific MTB survey, and a 5-level maturity scoring algorithm. The aim of this phase was to establish face validity and content validity through triangulation of the three sources of gathered insights. The model, survey, and scoring algorithm were developed to reflect the key processes of MTBs that apply fundamentally to all MTBs regardless of location. That is, the ACTE-MTB maturity model domains of Access, Consultation, Technology, and Evidence are fundamentally applicable to all MTBs and inherently have varying levels of complexities. Thus, face validity was established in Phase 1 since the 3 outputs appeared to be capable of grading MTB maturity in these domains. And because the outputs were believed to be reflective of all the relevant (and universal) MTB activities and processes required to foster a comprehensive MTB maturity measurement anywhere in the world, content validity was also established in this phase.

**Fig 2:**
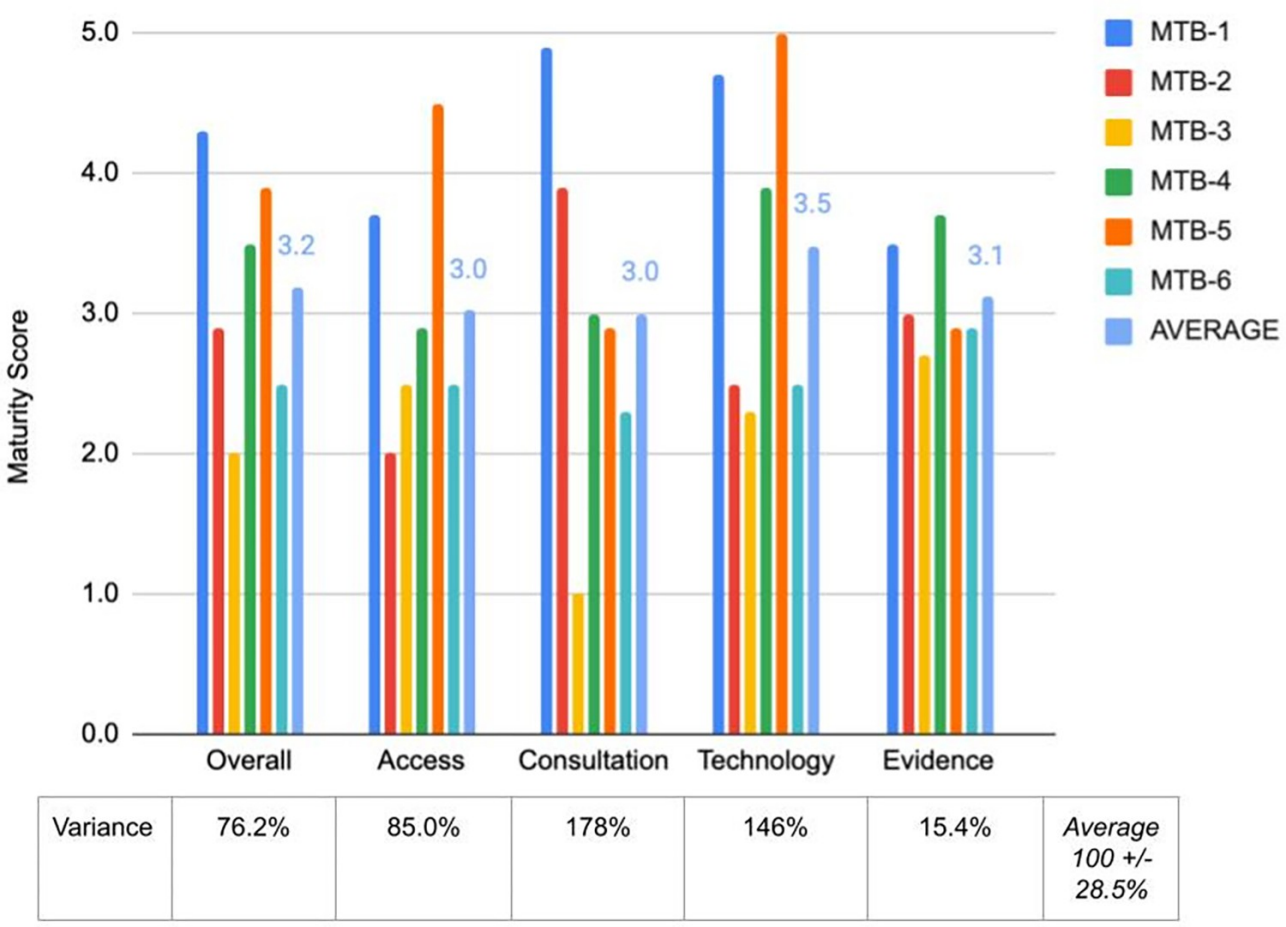
Overall and domain-specific maturity scores for 6 MTBs used for Phase 2 validity testing. MTBs 1–6 correspond to the 6 MTBs in the exact order presented in Table 2. Individual and average domain-specific and overall maturity scores were plotted on a scale from 0 to 5.0. % Variance from the mean is shown for the 6 maturity scores in each category and averaged across the categories with a standard error.

In Phase 2, the goal was to establish initial construct validity by demonstrating that the ACTE-MTB assessment tool was sensitive enough to generate different maturity scores across 6 different MTBs. These 6 MTBs were represented by mostly medical oncologists from two academic institutions, two specialized cancer centers, one private hospital, and one community hospital spanning Australia, China, Germany, and the United States (Table 2). ACTE-MTB assessments yielded average, overall, and domain-specific maturity scores among the 6 MTBs close to the middle of the scoring range, i.e., between 3.0 and 3.5 out of 5.0 (Fig 2). In addition, there was a non-zero degree of variance from the mean for overall and domain-specific maturity scores, averaging 100 +/- 28.5% (range: 15.4% - 178%), with the lowest degree of variance observed for the Evidence score and the highest observed for the Consultation score.

**Fig 3:**
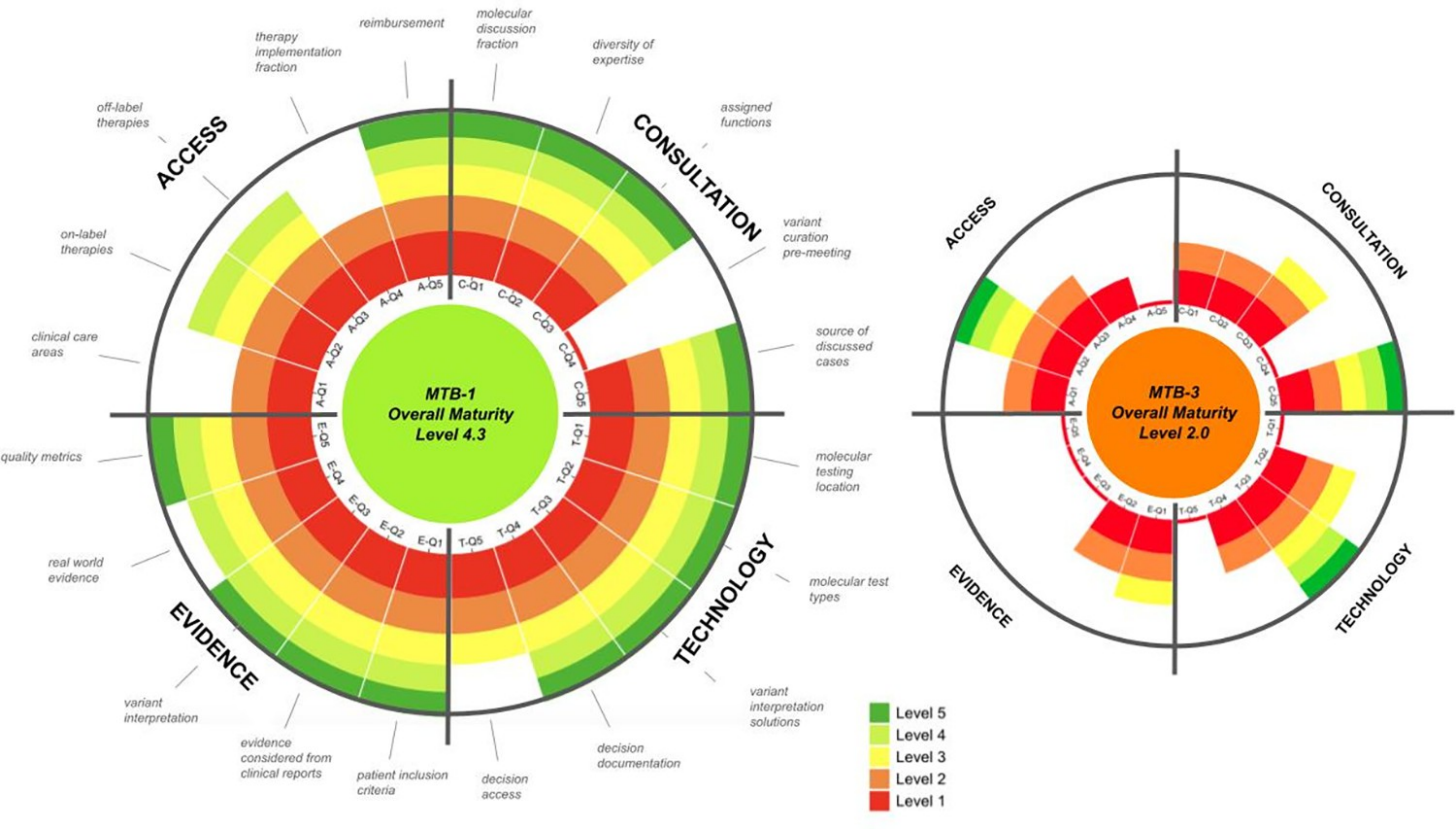
Radial plot of ACTE-MTB maturity assessments for highest and lowest maturity MTBs in Phase 2. Wedges in the radial plot represent individual survey questions with a small phrase provided at the top of each wedge to indicate the nature of the question. Each question is worth 5 points, with answers plotted on a “stoplight” color scale from Level 1 to 5. Overall maturity levels represent the average maturity score across the 4 categories, Access, Consultation, Technology, and Evidence.

The MTBs with highest overall and lowest overall maturity scores (MTB-1 and MTB-3, respectively) were plotted onto radial stacked bar charts to further illustrate the maturity differences between the MTBs at the level of each survey question, each ACTE domain, and overall (Fig 3). Taken together, these results suggest that the ACTE-MTB assessment tool is capable of discerning maturity score differences across different MTBs, thus establishing initial construct validity and paving the way to perform further, larger scale construct validity testing in Phase 3.

**Fig 4:**
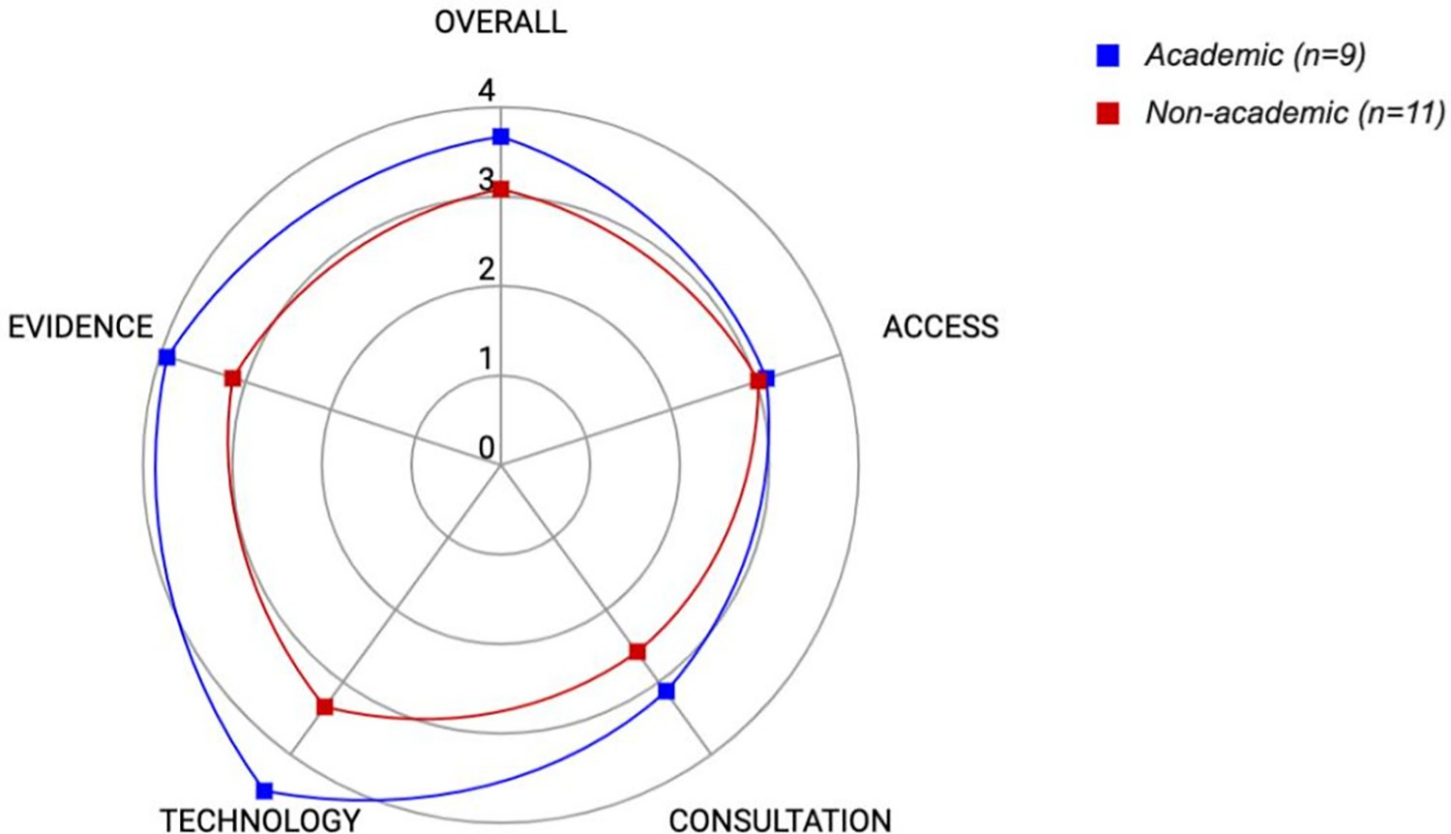
Comparison of averaged overall and domain maturity scores for academic vs. non-academic MTBs.

To further test construct validity of the ACTE-MTB tool, we began with the hypothesis that maturity levels would score higher for academic institutions as compared to non-academic institutions given the greater likelihood of academic institutions performing in-house molecular testing, supporting clinical trials, and carrying out research programs. Thus, the 6 MTBs from Phase 2 were combined with an additional 14 MTBs for a total of 20 MTBs. This allowed for a reasonable balance of academic (n = 9) vs. non-academic (n = 11) institution types undergoing the ACTE-MTB assessment. MTBs at academic institutions exhibited significantly higher overall maturity scores than MTBs in non-academic institutions (averaging 3.7 +/- 0.2 vs. 3.1 +/- 0.2, respectively; P = .018), supporting that the ACTE-MTB assessment tool can effectively differentiate between academic vs. non-academic MTBs (Table 4, Fig 4), and further establishing construct validity of the tool.

**Table 4:** ACTE-MTB maturity levels across 20 MTBs stratified by academic vs. non-academic institution type.

Institution Type	Country	Overall Maturity Level	Consultation	Evidence	Technology	Access	Therapy Implementation Rate
Academic	Australia	4.3	3.7	4.9	4.7	3.5	25%
Academic	Austria	4.3	4.5	4.3	4.9	3.7	75%
Academic	China	3.3	1.9	3.7	3.9	3.5	25%*
Academic	France	4.0	3.9	4.3	4.9	2.9	25%
Academic	France	3.3	2.5	4.0	3.9	2.5	50%
Academic	South Korea	3.0	1.3	3.9	4.3	2.9	100%
Academic	United Kingdom	3.5	3.0	3.9	4.5	2.9	25%*
Academic	United States	3.9	4.5	2.9	5.0	2.9	50%
Academic	United States	3.5	3.0	3.3	4.3	3.3	50%
**Academic MTB Averages**		**3.7 +/‒ 0.2**	**3.1 +/‒ 0.4**	**3.9 +/‒ 0.2**	**4.5 +/‒ 0.1**	**3.1 +/‒ 0.1**	**47.2 +/‒ 8.8%**
Community	Germany	2.0	2.5	1.0	2.3	2.7	25%
Community	Germany	2.7	1.7	2.3	2.5	4.0	100%
Community	Germany	3.9	2.0	5.0	4.3	3.3	75%
Community	United Kingdom	3.3	3.0	3.0	4.0	2.7	25%*
Private	Germany	3.5	2.9	3.0	3.9	3.7	50%
Private	United States	2.7	1.9	3.3	3.0	2.7	75%
Specialized center	China	2.9	2.0	3.9	2.5	3.0	50%
Specialized center	China	3.5	2.5	4.3	3.3	3.7	75%
Specialized center	China	3.5	3.5	4.7	4.0	2.0	25%
Specialized center	United Kingdom	3.3	3.9	1.9	4.3	2.7	25%*
Specialized center	United States	2.5	2.5	2.3	2.5	2.9	25%
**Non-Academic MTB Averages**		**3.1 +/‒ 0.2**	**2.6 +/‒ 0.2**	**3.2 +/‒ 0.4**	**3.3 +/‒ 0.2**	**3.0 +/‒ 0.2**	**50.0 +/‒ 8.3%**
**All MTB Averages**		**3.3 +/‒ 0.1**	**2.8 +/‒ 0.2**	**3.5 +/‒ 0.2**	**3.9 +/‒ 0.2**	**3.1 +/‒ 0.1**	**48.8 +/‒ 5.9%**

Average overall maturity levels were significantly higher for MTBs in academic institutions vs. other institution types (P = .018). Average therapy implementation rates were not significantly different between these two cohorts (P = .82). Averages are represented with a standard error range to accommodate small sample sizes. *Therapy implementation rates were estimated to be 25% for cases where respondents entered “I’m not sure.”

In performing construct validity testing, some interesting insights were uncovered. The higher overall maturity in academic MTBs compared to non-academic MTBs can be attributed to comparatively higher maturity scores in the Consultation, Technology, and Evidence categories. However, maturity scores for the Access category were virtually the same between the two groups. We therefore explored whether there was a correlation between Access maturity scores and the percentage of MTB-recommended therapies that are administered in patients (therapy implementation rate), since this is one of the scored survey questions of the Access category. Interestingly, across the 20 MTBs, we found an average reported therapy implementation rate of about 48.8 +/- 5.9% (Table 4). Further, the average therapy implementation rates between MTBs at academic centers and MTBs at non-academic centers were not statistically different (47.2 +/- 8.8% vs. 50.0 +/- 8.3%; P = .82). This suggests that while MTBs may score highly in operational aspects, the downstream therapy implementation impact does not correlate with overall maturity levels and thus represents a potential universal area of improvement for MTBs.

## Discussion

Comprehensive genomic profiling is becoming more routine in clinical practice as evidenced by professional guidelines incorporating recommendations for broad genomic profiling to identify molecularly-guided therapy options [13, 14]. In spite of this transition into routine clinical practice, there has been wide variability in actionable mutation rates (18–78%) and targeted treatment matching rates (6–70%) in prospective clinical trials that focus on multigene sequencing [15–19]. Actionable mutation assignment and molecularly-guided therapy matching is dependent on several inherently-variable factors, including classification methods, evidence levels, expertise, and the availability of therapies in a given region, which offers a plausible explanation for the variability seen in these studies. Molecular tumor boards focus on actionability and therapy matching and therefore present a forum on which to standardize these activities. The ACTE-MTB maturity assessment tool was developed with the aim to cultivate a comprehensive, structured view on the processes by which MTBs operate and ultimately reach decisions on molecularly-guided diagnosis and/or therapy plans and to provide a foundation for the development of defined standards and best practices for MTB activities. In the process of developing the tool and performing validity testing, there were several learnings, which are highlighted below.

In Phase 1 development of the ACTE-MTB tool, both face and content validity were established and reflected through the 3 outputs: 1) the ACTE-MTB maturity model; 2) a 59-question survey; and 3) a 5-level maturity scoring algorithm. In the process of carrying out Phase 1, there were several key learnings and considerations. First, it was clear from insights-gathering stages that there were varied understandings of the definition of “MTB.” These understandings can be summarized into 2 categories where an MTB is: 1) A specialized, dedicated team or forum that focuses on analyzing and discussing only molecular data as part of a patient’s care pathway; or 2) Any MDT or tumor board that incorporates molecular data into the broader discussion. To avoid limitations of basing the ACTE-MTB tool on a single definition, the survey was designed to ask whether participants believed they actively participated in an MTB and if so, to specify the proportion of patient cases that had molecular data discussed. If a respondent answered 100%, then it was assumed that they participated in an MTB consistent with the first definition, while all other responses assumed the 2nd definition. All 20 respondents for this study believed they participated in MTBs, but interestingly, the reported proportion of molecular data that was discussed varied widely across respondents (from <25% to 100% and averaging 50%), further confirming the different understandings of the definition of an MTB and highlighting an area in need of clarification across the field. This could certainly be achieved as part of MTB guidelines and best practices development.

Second, with regard to the ACTE-MTB model, clearly the “house” representation that was presented in Fig 1 is high level and does not capture important nuances in each of the domains. The intent was for the model to provide a digestible framework with the potential for expansion to achieve greater granularity in both the pillars and in the individual blocks that make up the pillars. For example, the Evidence pillar can naturally be expanded to a related sub-pillar of Actionability Classifications [20, 21], which ranks the quality of evidence supporting each individual component of the pillar (S2 Fig). This figure further illustrates expansion of an individual block, for example, the Real-World Evidence (RWE) block, which is further classified into sources and types of RWE. This horizontal and vertical expansion strategy coupled with the structured 59-question MTB survey fosters a more granular assessment of MTB capabilities, infrastructure, and processes for more accurate, relatable results and richer discussions with MTB participants.

Third, the MTB maturity scoring algorithm was intentionally designed to allow for room to improve i.e. it was calibrated to the maturity levels shown in the ACTE-MTB model, where most MTBs were assumed *a priori* to be at about a Level 3 out of 5. And this bore out in later phases of testing where average overall maturity scores across the 20 MTBs were around 3.3. In other words, if all MTBs initially scored at a 4 or above, there would not be much room to improve. Also, generally high initial baseline maturities across MTBs assessed with the ACTE-MTB tool in this study would not be consistent with the insights gathered in Phase 1, which highlighted a plethora of challenges MTBs are currently facing in each of the four ACTE domains.

In Phases 2 and 3, construct validity of the ACTE-MTB tool was established. In Phase 2, we applied the ACTE-MTB tool in the real-world setting, on 6 MTBs in Australia, China, Germany, and the United States and spanning all 4 institution types (academic, specialized, private, and community). We initially established that the tool can be used to measure maturity of different MTBs regardless of location and institution type. Averaged overall and domain-specific maturity scores landed where we had hoped when developing the algorithm in Phase 1, around 3.0. Thus, initial construct validity was established. The tool was then applied in Phase 3 in a wider context, as an extension of Phase 2, to test academic vs. non-academic differences in maturities. Here, statistically significant maturity score differences for each group were observed, thus further establishing construct validity. In performing validity testing, there was no standard or criterion in the field on which to benchmark the results; however, given the variance in scores observed across the 20 MTBs (Figs 2 and 4, Table 4), it can be concluded that ACTE-MTB is capable of discriminating between functionally-different MTBs, and, thus, is well-poised for providing insights from larger scale assessments.

In the process of running validity testing in Phase 3, some interesting insights were uncovered around the Access category, which deserve further exploration. Access maturity scores were lower than Consultation, Technology, and Evidence maturity scores and not significantly different between academic and non-academic institutions (Table 4, Fig 4). One of the components of the Access maturity score is the rate at which therapies recommended by MTBs are implemented. In looking more closely at survey responses for this question, across the 20 MTBs, it was reported that only about 50% of therapies recommended by the MTBs are actually implemented. Of note, there was significant variance in therapy implementation rates reported by respondents, ranging from 25% to 100%. The exact cause for this variance remains unclear, but possible explanations include, firstly, that this therapy implementation rate may have been an estimate for many of the MTBs. Less than half of the survey respondents reported capturing implemented treatments in a structured format (database) which may have resulted in an inaccurate calculation of the true therapy implementation rate. Secondly, this variance could be attributed to the fact that the survey question relevant to therapy implementation rate did not differentiate between the different categories of treatments recommended by the MTBs, e.g., novel/early phase therapeutics, targeted medications, standard-of-care therapies, or even no therapies. Regardless, this highlights a universal area of need, requiring further investigation on a larger scale. Follow-up with the survey respondents is underway to understand the nuances of therapy implementation in these MTBs. Capturing the therapy implementation rate specifically for non-standard-of-care treatment approaches, as well as the percentage of these approaches accessed via clinical trials, compassionate-use programs, and self-funding will provide important insights around this aspect.

There was an *a priori* expectation that therapy implementation rates would correlate with significantly higher overall maturity levels in academic centers vs. other institution types given a higher likelihood of academic centers’ access to early phase clinical trials, dedicated funding for treatment arms attached to the MTB, and experience with accessing emerging non-government-agency-approved medications. However, therapy implementation rates were not significantly higher in MTBs housed in academic centers, suggesting that additional factors and/or barriers may play a role. Presumably, therapy implementation rates would go beyond 50% if there were balanced recommendations based on levels of evidence, standardization, citation of evidence supporting decisions, and clinical-trial-directed referrals. All of these elements ultimately aim to reduce the cost of drug access pathways, with clinical trial enrollment being a preferred pathway, owing to its high cost-effectiveness and potential to lower risk to hospitals using off-label medications.

About half of the survey respondents cited that the reason for low therapy implementation rates were issues with access to the medications. This was often due to the unavailability of medications at a particular institution, lack of trial availability, and denied pre-authorizations and claims by payers. Additionally, just under half of the survey respondents cited that the reason was because the patient died before therapy could be started. This highlights a key issue regarding appropriate patient selection for MTB assessment and potentially the often-prolonged time periods awaiting the results from molecular testing. The survey asked the approximate number of weeks between beginning the process of molecular testing to discussing the results in the MTB, and thereafter, to starting targeted therapy. Across the 20 respondents, the average time from start of molecular testing to start of therapy was 8.5 +/- 0.7 weeks. This highlights a key potential area for deeper investigation into the rate-limiting steps for each MTB and the nuances behind these steps that would likely benefit from one or more process improvements, which could include referring patients to MTBs at much earlier time points in their disease process.

It is interesting to also consider that the discrepancy in maturity scores seen between Access and the other categories represents the limits to which a single organization can impact the adoption of MTBs. That is, organizations can evolve their internal process, invest in technologies to enhance them, and apply the latest knowledge, but are limited at the organizational level when it comes to access to testing and/or the latest therapies, indicating that this is a system-level issue. Thus, in the ACTE-MTB tool, the A of ACTE ultimately measures “system readiness” while the C, T, and E measure “provider readiness.” One application of the ACTE-MTB tool and its findings could be to frame conversations between organizations and the overarching systems within which they operate to identify the key hurdles to having successful realization of MTB recommendations.

The ACTE-MTB maturity assessment tool offers three main advantages: 1. Guidance and considerations around what is needed to implement and improve MTBs i.e., a blueprint; 2. A view into similarities and differences across different MTBs, providing a framework for global MTB standards and best practices definition; and 3. A benchmarking tool to assess maturity of an MTB at a given point in time and perform score comparisons over time to track the impact of making process changes. As part of any standardization process, quality management is necessary not only to ensure best practices, but to also inform and guide improvements to the current standards. ACTE-MTB was adapted from the CMMI model, a proven framework for benchmarking quality and determining process improvements in organizations delivering products and services [3]. While the CMMI model focuses on managing quality through process improvements, this study did not focus on this aspect and instead focused on maturity measurement. However, it is envisioned that process improvements are the natural next step after measuring the maturity of MTBs. To this end, the color-coded radial plots of maturity assessments easily highlight areas for development and foster structured follow-up discussions with the assessed MTBs, not only to understand nuances, but also to collaborate on potential process improvements. Of note, the use of radial plots to represent areas in healthcare needing improvement and for tracking progress is not novel and has been previously demonstrated in the oncology field [4]. Using radial plots to demonstrate the level of ACTE-MTB maturity as a whole and for each domain facilitates the assessment of a given institution, while also providing guidance on the specific domains, or areas within each domain that are subject to improvement opportunities. Further, this application can help emphasize critical areas when considering development, implementation or simply for benchmarking MTBs across institutions. Future, larger scale validation of this model will help solidify these functionalities.

It is important to note that in this study of 20 MTBs, there were sources of variability that could potentially affect the results of the ACTE-MTB scoring. First, only a single individual representing each MTB was queried and therefore the impressions of the MTB may be skewed by a single opinion. In addition, multiple, different roles were queried and this introduces the additional variable of role-based impressions. Assessments of MTBs are currently underway which query multiple roles in a single MTB, and in making comparisons, it will be critical to do 2 things: 1) Get a consolidated view of the single MTB; 2) When making comparisons across MTBs, to leverage the consolidated view as well as make role-specific comparisons. Second, variability may have been introduced by surveying MTBs that focus on different cancer types. Some were focused on a single cancer, others taking more of a pan-cancer approach. One might imagine that MTBs that more often discuss cancers with molecular testing and targeted therapies well-represented in clinical practice guidelines would have a very different ACTE-MTB profile than MTBs that more often discuss rarer cancers with less defined and accepted precision medicine pathways.

In developing the ACTE-MTB maturity assessment tool and applying it to 20 MTBs from different institution types around the globe, specific areas emerged across the 4 pillar categories which we believe represent key considerations for all MTBs. For each ACTE-MTB category, there are 5 considerations (Table 5) which align with the 5 maturity aspects (wedges) depicted in the radial plots in Fig 3. To summarize these considerations holistically, in the Access category, MTBs should endeavor for all patients to undergo molecular profiling, in place of the current *status quo* which prioritizes just those with late-stage or treatment-refractory conditions. Following sequencing, MTBs should set out to have programs in place to facilitate the use of off-label, emerging, and compassionate-use therapies, to foster a therapy implementation rate to as close to 100% as possible. In the Consultation category, it is essential to have a dedicated MTB with a diverse set of expertise (including external experts) where 100% of the cases have molecular data discussed. In the Technology category, it is prudent to have solutions that ensure multiple molecular testing results and evidence can be rigorously deliberated and that MTB decisions can be documented and accessed by all participants of the group. In the Evidence category, it is critical to venture beyond guidelines (clinical / preclinical studies, clinical trials, real-world evidence, etc.) in making molecularly-guided therapy decisions and to consider both somatic and germline-supporting evidence. Because these considerations hold applicability for all MTBs, we believe they provide a foundation for creation of global MTB guidelines.

**Table 5:** Key MTB considerations for global MTB best practices.

**ACCESS**	**CONSULTATION**
• MTB decisions should influence more than the care tied to determination of therapies for late-stage or relapsed patients and should be threaded through the entire care continuum. Ultimately, every patient should be sequenced as part of their diagnostic work-up. • MTBs should have programs in place to facilitate the use of off-label, emerging, and compassionate-use therapies. • MTBs should have robust links and referral pathways in place for enrollment into clinical trials. • MTB therapy recommendations should be implemented in as many patients as possible. • MTBs should have codes tied to reimbursement and should exert their influence beyond the MTB and drive local/national policy for access to therapies.	• 100% cases in the multidisciplinary meeting should have molecular data discussed as part of the case presentation. • There should be a diverse attendee profile represented in the molecular tumor board to cover clinical and scientific discussions, including representatives of oncology, pathology, pharmacy, clinical genetics, bioinformatics, and translational research. • The MTB activities should be assigned and spread evenly and appropriately across the multiple roles to foster shared accountability and sustainability. • For MTBs discussing results of in-house testing, a variant curation “pre-meeting” is recommended to ensure efficiency in the MTB meeting. • Inclusion of external clinicians and scientists is encouraged to provide richer perspectives and expertise.
**TECHNOLOGY**	**EVIDENCE**
• MTBs that occur in institutions running their own sequencing (in-house) have control over the sequencing data and can go beyond clinical practice and into the research realm, which fosters proactive discovery. • MTBs should strive to have a holistic molecular view going beyond NGS and incorporating other modes of molecular testing, including RNA-based, and protein-based approaches. • MTBs should have a platform on which all the salient data and clinical information are brought together to facilitate variant interpretation, discussions and decision-making. • MTB decisions should be electronically documented for referral and easy access post the meeting. • Documented MTB decisions should be readily available to all MTB participants, including external stakeholders.	• All patients should get a comprehensive genomic profile at any stage of any cancer. This may not only identify potential first line targeted therapy options but serves as a baseline for monitoring progression. • MTBs should consider as much relevant evidence as possible to make decisions. This includes evidence beyond clinical practice guidelines (e.g. clinical and preclinical studies, hereditary/germline evidence, and real world evidence). • All parties of the MTB should have a level of proficiency in variant interpretation consistent with an understanding of the professional society guidelines for somatic and germline variant interpretation, given this aspect is foundational to determining actionability. • Capturing and leveraging Real World Evidence is encouraged and may include tracking outcomes, bolstering insurance pre-authorization evidence claims, and performing patient similarity analytics. • MTB quality should be consistently tracked and should include statistics on molecular testing, identification of actionable mutations, matched therapies, therapies implemented, and outcomes.

## Conclusions and next steps

To our knowledge, ACTE-MTB is the first tool of its kind to enable structured and universal maturity assessments of MTBs. Validity testing demonstrated feasibility for appropriate domain-specific assessment and confirmed its ability to differentiate between MTBs housed in functionally-different types of institutions. A larger-scale, global, assessment using the ACTE-MTB tool is currently underway to confirm findings in this preliminary set of 20 MTBs and to uncover new, significant findings. Next, we aim to pilot the ACTE-MTB assessment tool at institutions and further develop it into an interactive platform which enables MTBs to systematically self-analyze their status and identify opportunities for continuous improvement. Finally, we believe the findings presented in this publication can serve as a foundation for the development of professional societal guidelines and best practices for MTBs, which would result in increased uptake of MTBs, enhanced molecularly-guided therapy matching frameworks, and improved patient outcomes.

## Supporting information

S1 Fig Maturity level scale. Five 5-point questions per ACTE domain, for a possible total of 25 points, were evenly distributed across maturity levels 1–5.
(TIF)

S2 Fig Example of vertical and horizontal drill-down of evidence components of ACTE-MTB. Actionability classifications are based on guidelines from the professional societies, AMP (Association for Molecular Pathology)/ASCO (American Society of Clinical Oncology)/CAP (College of American Pathologists) [20] and ESMO (European Society for Medical Oncology) [21]. **RWE**: Real World Evidence; **EHR**: Electronic Health Record; **Tx**: Treatment/Therapy; **PRO**: Patient Reported Outcome; **VUS**: Variant of Uncertain Significance; **ESCAT**: ESMO Scale for Clinical Actionability of molecular Targets [21].
(TIF)

S1 Table Examples of translated survey answers to points values that feed maturity scores. (TIF)

S1 File Minimal dataset. This dataset contains the translation of survey responses to points and maturity levels as well as statistical calculations used throughout the study.
(XLSX)

S2 File MTB scientific survey. The complete 59-question survey administered online to 20 participants between January and July 2021.
(PDF)
