# Supplementary figures and images for: Development and validation of ACTE-MTB: A tool to systematically assess the maturity of molecular tumor boards
Source: PLoS One. 2022 May 13;17(5):e0268477. doi: 10.1371/journal.pone.0268477 (PMC9106161; doi:10.1371/journal.pone.0268477)

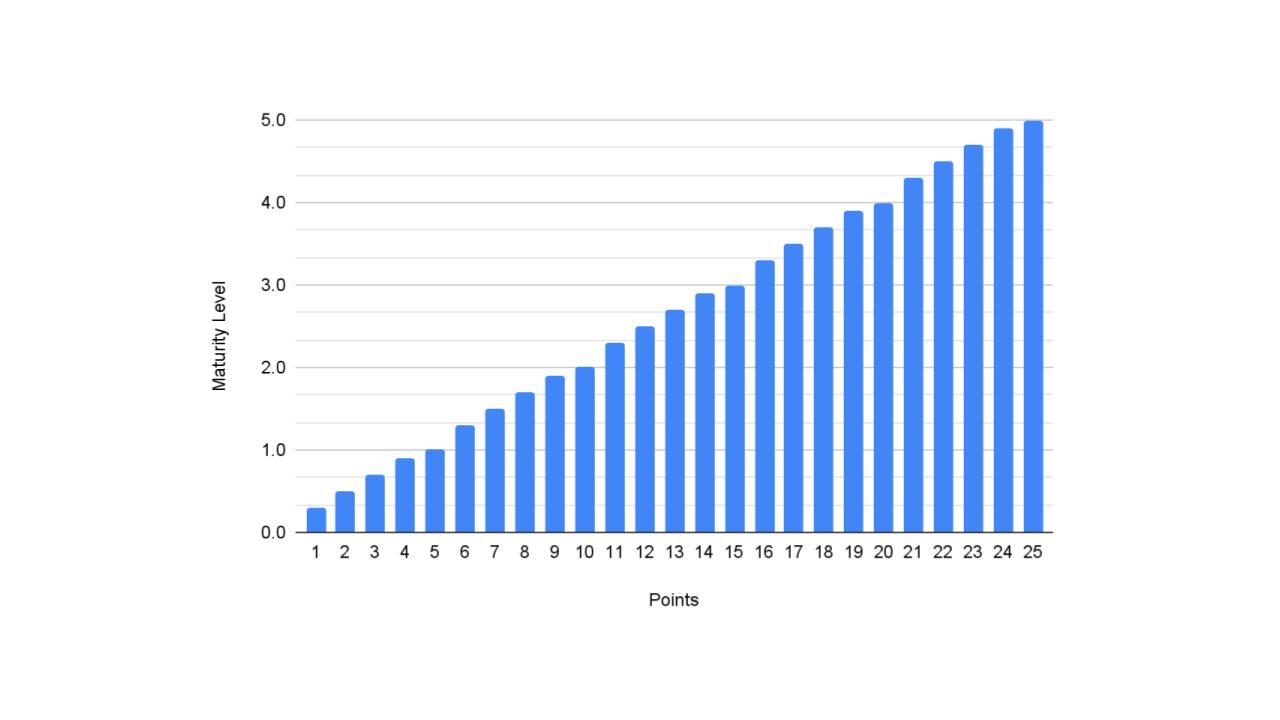

Supplement: S1 Fig — Five 5-point questions per ACTE domain, for a possible total of 25 points, were evenly distributed across maturity levels 1–5. (TIF) [file pone.0268477.s001.tif]

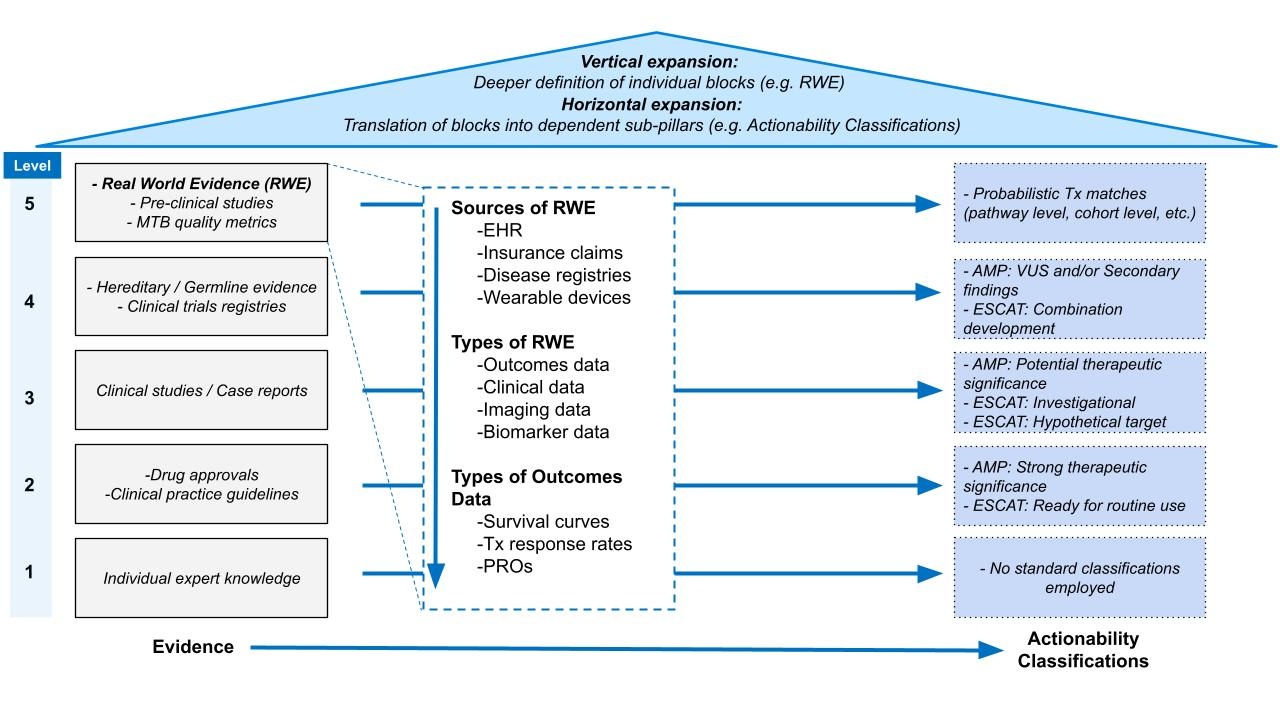

Supplement: S2 Fig — Actionability classifications are based on guidelines from the professional societies, AMP (Association for Molecular Pathology)/ASCO (American Society of Clinical Oncology)/CAP (College of American Pathologists) [20] and ESMO (European Society for Medical Oncology) [21]. RWE: Real World Evidence; EHR: Electronic Health Record; Tx: Treatment/Therapy; PRO: Patient Reported Outcome; VUS: Variant of Uncertain Significance; ESCAT: ESMO Scale for Clinical Actionability of molecular Targets [21]. (TIF) [file pone.0268477.s002.tif]

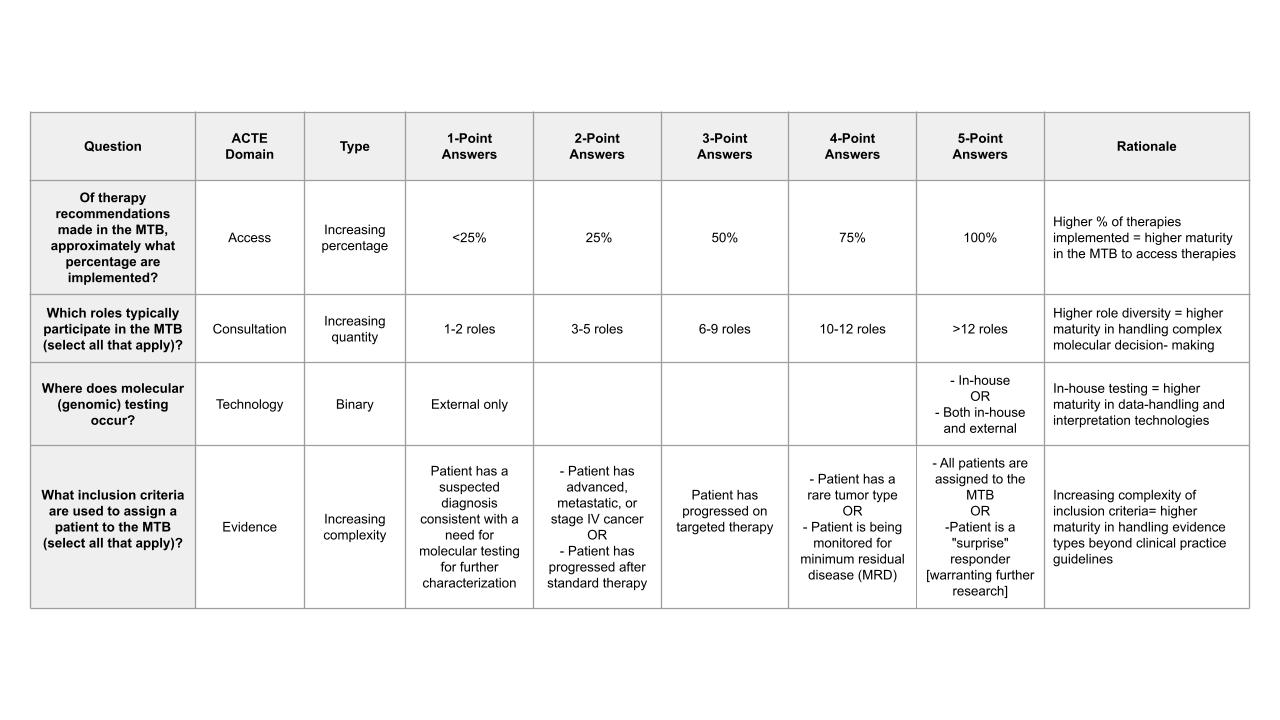

Supplement: S1 Table — (TIF) [file pone.0268477.s003.tif]
