# Supplementary material for: Development and validation of ACTE-MTB: A tool to systematically assess the maturity of molecular tumor boards
Source: PLoS One. 2022 May 13;17(5):e0268477. doi: 10.1371/journal.pone.0268477 (PMC9106161; doi:10.1371/journal.pone.0268477)
Supplement: S2 File — The complete 59-question survey administered online to 20 participants between January and July 2021. (PDF) [file pone.0268477.s005.pdf]

Molecular Tumor Board (MTB) Scientific Survey

This global scientific survey assesses various aspects of health care provider impressions of their molecular tumor board (MTB) program, including aspects of molecular testing, discussion topics, operations, and evidence utilization. It takes approximately 30 minutes to complete and your participation is voluntary.

The data generated from this survey will be anonymized and strictly used to identify trends in MTB programs to better understand similarities and differences in MTBs across the globe. In the event the data from this survey are published, institution names and interviewee names will NOT be disclosed; however, type of institution and country may be disclosed (e.g. Academic Medical Center, USA) as a method to stratify global trends.

All questions are aimed at understanding YOUR experience, views, and opinions vs. those of your institution. In the last survey question, there is an opportunity to provide your email address only if you choose to be contacted for a brief follow-up discussion. Your participation in this survey will otherwise remain completely anonymous. In your responses, please do not include any confidential or proprietary information about you, your institution, or patients. Thank you for taking this survey!

---

* Required

Demographics

1. 1. Date *

____

Example: January 7, 2019

2. 2. Location (City / State / Country) *

____

3. 3. Institution Setting (select all that apply) *

Check all that apply.

- [ ] Academic medical center or research institute
- [ ] Community or rural hospital
- [ ] Specialized cancer clinic
- [ ] Office / Private Practice

Other: [ ] _______________________________

4. 4. Institution Type (select all that apply) *

Check all that apply.

- [ ] Single institution
- [ ] Core or "hub" of a network
- [ ] Satellite or "spoke" of a network
- [ ] MTB service provider

Other: [ ] ______________________

5. 5. Your Role (select all that apply) *

Check all that apply.

- [ ] Medical Oncologist
- [ ] Surgical Oncologist
- [ ] Radiation Oncologist
- [ ] Radiologist
- [ ] Anatomical Pathologist
- [ ] Molecular Pathologist
- [ ] Physician/Organ Specialist
- [ ] Nurse Navigator
- [ ] Registry Nurse / Registrar
- [ ] Pharmacist
- [ ] Bioinformatician / Clinical Informatician
- [ ] Human or Clinical Geneticist / Genomicist
- [ ] Genetic Counselor
- [ ] Research Scientist / Clinical Scientist / Molecular Biologist / Translational Scientist
- [ ] Data Manager
- [ ] Laboratory Specialist
- [ ] MTB Coordinator
- [ ] C-level Executive (CEO, CMO, CFO, CIO, etc)

Other: [ ] ______________________

6. 6. Approximately how many NEW oncology patients per year do you treat? *

Mark only one oval.

- Less than 25
- 25-50
- 50-100
- 100-200
- More than 200
- I'm not sure
- N/A

7. 7. For which cancer types do you make clinical decisions (select all that apply)? Please use the "Other" option to specify any types not already listed. *

Check all that apply.

- [ ] Breast
- [ ] Digestive / GI
- [ ] Endocrine / Neuroendocrine
- [ ] Gynecologic
- [ ] Genitourinary
- [ ] Head and Neck
- [ ] Hematologic / Blood
- [ ] Musculoskeletal
- [ ] Neurologic
- [ ] Skin / Soft Tissue
- [ ] Thoracic / Respiratory
- [ ] Unknown Primary
- [ ] Pediatric cancers
- [ ] Rare cancers

Other: [ ] ______________________

8. 8. Are you actively involved in making clinical decisions based on molecular testing (whether directly or in a supporting capacity)? *

Mark only one oval.

- Yes
- No

9. 9. Do you participate in multidisciplinary team (MDT) or tumor board (TB) meetings? *

Mark only one oval.

- Yes
- No

10. 10. If yes, approximately what percentage of patients have molecular / genomic information discussed as part of their case presentation? *

Mark only one oval.

- None
- Less than 25%
- 25%
- 50%
- 75%
- 100%
- I'm not sure
- N/A

11. 11. Would you define or classify any of the MDT / TB meetings (in which you are participating) as "Molecular Tumor Board" meetings? *

Mark only one oval.

- Yes *Skip to question 12*
- No *Skip to question 57*

MTB Operations

12. 12. How many participants typically attend the MTB meeting? *

Mark only one oval.

- ⬭ Less than 5
- ⬭ 5-10
- ⬭ 10-15
- ⬭ 15-25
- ⬭ 25-40
- ⬭ >40
- ⬭ I'm not sure

13. 13. Which roles typically participate in the MTB (select all that apply)? *

Check all that apply.

- [ ] Medical Oncologist
- [ ] Surgical Oncologist
- [ ] Radiation Oncologist
- [ ] Radiologist
- [ ] Anatomical Pathologist
- [ ] Molecular Pathologist
- [ ] Physician/Organ Specialist
- [ ] External Clinician or Scientist
- [ ] Nurse Navigator
- [ ] Registry Nurse / Registrar
- [ ] Pharmacist
- [ ] Bioinformatician / Clinical Informatician
- [ ] Human Geneticist / Clinical Geneticist
- [ ] Genetic Counselor
- [ ] Research Scientist / Clinical Scientist / Molecular Biologist / Translational Scientist
- [ ] Data Manager
- [ ] Laboratory Specialist
- [ ] MTB Coordinator
- [ ] C-level Executive (CEO, CMO, CFO, CIO, etc)

Other: [ ] ______________________

14. 14. If external clinicians are participating in the MTB, from which types of institutions are they coming (select all that apply)?

Check all that apply.

- [ ] Academic medical center or research institute
- [ ] Community or rural hospital
- [ ] Specialized cancer clinic
- [ ] Office / Private Practice

Other: [ ] ______________________

15. 15. If your MTB has assigned functions, which role(s) perform the listed functions (select all that apply)?

Check all that apply.

	Gathers data	Interprets data	Presents data	Moderates the discussion	Documents the recommendations	Manages follow-up activities (e.g. monitoring)
Oncologist	[ ]	[ ]	[ ]	[ ]	[ ]	[ ]
Pathologist	[ ]	[ ]	[ ]	[ ]	[ ]	[ ]
Physician / Organ Specialist	[ ]	[ ]	[ ]	[ ]	[ ]	[ ]
Scientist(s) (bioinformatician, geneticist, etc)	[ ]	[ ]	[ ]	[ ]	[ ]	[ ]
Nurse(s)	[ ]	[ ]	[ ]	[ ]	[ ]	[ ]
MTB Coordinator	[ ]	[ ]	[ ]	[ ]	[ ]	[ ]

16. 16. What types of incentives, if any, are given to participants in the MTB (select all that apply)? *

Check all that apply.

- [ ] Monetary compensation
- [ ] Educational credits towards licensure (e.g. CMEs)
- [ ] Authorship on publications that may emerge from MTB findings
- [ ] None
- [ ] I'm not sure

Other: [ ] ____________________

17. 17. How often does the MTB meet? *

Mark only one oval.

- ( ) Daily
- ( ) Weekly
- ( ) Bi-weekly
- ( ) Monthly
- ( ) Quarterly
- ( ) Ad hoc (when needed)

18. 18. What is the typical length of a meeting? *

Mark only one oval.

- ( ) 0.5-1 hour
- ( ) 1-2 hours
- ( ) >2 hours

19. 19. Is there a separate, dedicated "curation" meeting ahead of the MTB meeting where molecular alterations are vetted and prioritized? *

Mark only one oval.

- ( ) Yes
- ( ) No
- ( ) I'm not sure

MTB Patients

20. 20. What are the most common cancers discussed in the MTB (select all that apply)? Please use the "Other" option to specify any types not already listed. *

Check all that apply.

- [ ] Breast
- [ ] Digestive / GI
- [ ] Endocrine / Neuroendocrine
- [ ] Gynecologic
- [ ] Genitourinary
- [ ] Head and Neck
- [ ] Hematologic / Blood
- [ ] Musculoskeletal
- [ ] Neurologic
- [ ] Skin / Soft Tissue
- [ ] Thoracic / Respiratory
- [ ] Unknown Primary
- [ ] Pediatric cancers
- [ ] Rare cancers

Other: [ ] ____________________

21. 21. What is the typical number of patients discussed in each meeting? *

Mark only one oval.

- ( ) Less than 5 patients
- ( ) 5-10 patients
- ( ) 10-15 patients
- ( ) 15-20 patients
- ( ) 20-30 patients
- ( ) More than 30 patients

22. 22. Do you see the number of patients increasing significantly in a year's time? *

Mark only one oval.

- Yes
- No
- I'm not sure

23. 23. What is the source of patients discussed at the MTB (select all that apply)? *

Check all that apply.

- Internal patients
- External patients (referred from outside the institution)

Other: ____________________

24. 24. What inclusion criteria are used to assign a patient to the MTB (select all that apply)? *

Check all that apply.

- All patients are automatically assigned to the MTB
- Patient has advanced, metastatic, or stage IV cancer
- Patient has a suspected diagnosis consistent with a need for molecular testing for further characterization
- Patient has progressed after standard therapy
- Patient has progressed and/or has acquired resistance to targeted therapy
- Patient is being monitored for minimum residual disease (MRD)
- Patient has a rare tumor type
- Patient is a "surprise responder"

Other: ____________________

25. 25. What areas of clinical care are influenced by the MTB decisions (select all that apply)? *

Check all that apply.

- Diagnosis
- First line therapy
- Therapy post progression or relapse
- Monitoring treatment response
- Follow-up (e.g. survivorship care)
- Supportive care (counseling- nutrition, social/mental, palliative care, etc.)

Other: ____________________

26. 26. For therapy decisions, approximately what fraction of each therapy type is recommended by the MTB? *

Mark only one oval per row.

	>80%	20-80%	<20%	None
Standard systemic therapies	◯	◯	◯	◯
Off-label systemic therapies	◯	◯	◯	◯
Compassionate-use systemic therapies	◯	◯	◯	◯
Non-systemic therapies (e.g. radiation, surgery)	◯	◯	◯	◯
No therapy due to recommendation to do more testing first	◯	◯	◯	◯

27. 27. If applicable, in what scenarios are patients discussed again at one or more subsequent MTB meetings (select all that apply)? *

Check all that apply.

- [ ] N/A- Patients are only usually discussed in a single MTB meeting.
- [ ] Patient case complexity requires additional discussion (e.g. additional testing required)
- [ ] Relapse
- [ ] Progression
- [ ] Another primary cancer has been identified

Other: [ ] ______________________

Molecular Testing

28. 28. Where does molecular (genomic) testing occur? *

Mark only one oval.

- ◯ In-house
- ◯ Externally (service provider)
- ◯ Both
- ◯ Other: ______________________

29. 29. If both, approximately what fraction of testing occurs in-house vs. externally? *

Mark only one oval.

- N/A
- Mostly or all in-house
- Mostly or all external
- Mix of both
- I'm not sure
- Other: ______________________

30. 30. Approximately what portion of molecular tests discussed are laboratory-developed tests (LDTs) vs. regulated in-vitro diagnostic (IVD) tests? *

Mark only one oval.

- Mostly or all LDT
- Mostly or all IVD
- Mix of both
- I'm not sure
- N/A

31. 31. What types of molecular tests are being discussed in the MTB (select all that apply)? *

Check all that apply.

- [ ] NGS DNA sequencing panels
- [ ] Circulating tumor DNA (ctDNA) tests
- [ ] PCR-based (e.g. for specific alterations like EGFR exon 19 deletions, KRAS G12C, etc.)
- [ ] Whole Exome Sequencing (WES)
- [ ] Whole Genome Sequencing (WGS)
- [ ] Sanger Sequencing
- [ ] Immunohistochemistry (IHC)
- [ ] Fluorescence In Situ Hybridization (FISH)
- [ ] Comparative Genomic Hybridization (CGH)
- [ ] mRNA expression assays
- [ ] RNASeq (e.g. fusion detection)
- [ ] Chromosomal Microarray Analysis (CMA)
- [ ] Multiplex Ligation-dependent Probe Amplification (MLPA)
- [ ] None
- [ ] I'm not sure

Other: [ ] ____________________

32. 32. Is the ordering of molecular tests usually decided in the MTB? *

Mark only one oval.

- ( ) Yes
- ( ) No, testing has usually already occurred and molecular information is available during the MTB discussion
- ( ) I'm not sure
- ( ) Other: ____________________

33. 33. What evidence (from molecular testing reports or otherwise) is used to prioritize molecular alterations for discussion in the MTB (select all that apply)? *

Check all that apply.

- [ ] Presence of "standard" approved therapies (whether by gov't agencies or endorsed in clinical practice guidelines)
- [ ] Presence of "off-label" approved therapies (indicated in a cancer type other than the patient's cancer type)
- [ ] Open, enrolling clinical trials
- [ ] Presence of emerging therapies (from preclinical studies)
- [ ] Prognostic predictions (e.g. validated risk scores)
- [ ] Diagnostic predictions (e.g. clarification of cancer type or sub-type)
- [ ] Potential hereditary disease association(s), including hereditary cancers
- [ ] Functional and biological significance of the genes / alterations
- [ ] Variant allele frequencies (somatic vs. germline)
- [ ] Variant classification categories (e.g. Tier 1, Pathogenic, etc.)
- [ ] Variant combinations with potential clinical impact

Other: [ ] ____________________

34. 34. Do you participate in the interpretation of data derived from molecular (genomic) testing? If yes, the next section will ask a few detailed questions on how alterations are interpreted. *

Mark only one oval.

- ( ) Yes *Skip to question 35*
- ( ) No *Skip to question 43*
- ( ) Other: ____________________

Skip to question 43

Molecular Alteration Interpretation

35. 34A. Which, if any, tiering or classification methods are utilized to interpret alterations? (select all that apply)? *

Check all that apply.

- [ ] AMP/ASCO/CAP actionability classification
- [ ] ESCAT (ESMO) actionability classification
- [ ] NCT (Germany) actionability classification
- [ ] MSKCC actionability classification
- [ ] ACMG pathogenicity classifications
- [ ] ACGS pathogenicity classifications
- [ ] In-house classifications (devised by the institution)
- [ ] None
- [ ] I'm not sure

Other: [ ] ______________________________

36. 34B. Which alteration categories are reported (select all that apply)? *

Check all that apply.

- [ ] Actionable alterations
- [ ] Variants of Unknown Significance (VUS)
- [ ] Variants associated with secondary (incidental) findings in hereditary cancer syndromes
- [ ] Variants associated with secondary (incidental) findings in hereditary diseases (other than cancer)
- [ ] I'm not sure

Other: [ ] ______________________________

37. 34C. Which alteration types are reported (select all that apply)? *

Check all that apply.

- [ ] Single nucleotide variants (SNVs)
- [ ] Small indels
- [ ] Large indels
- [ ] Copy number gain
- [ ] Copy number loss
- [ ] Fusions
- [ ] Splice site mutations
- [ ] Frameshift / truncating mutations
- [ ] Tumor mutational burden (TMB)
- [ ] Microsatellite instability (MSI)
- [ ] I'm not sure

Other: [ ] ______________________________

38. 34D. What evidence is used for matching alterations to therapies (select all that apply)? *

Check all that apply.

- [ ] Drug labels (FDA, EMA, CFDA, etc.)
- [ ] Clinical Practice Guidelines (e.g. NCCN, ESMO, KSMO, Regional Guidelines, etc.)
- [ ] Clinical Trials Databases (e.g. clinicaltrials.gov)
- [ ] Publications: clinical trials outcomes studies
- [ ] Publications: preclinical studies
- [ ] Professional society abstracts (e.g. ASCO, ESMO abstracts)
- [ ] Outcomes from similar patients
- [ ] I'm not sure

Other: [ ] ______________________

39. 34E. What sources are consulted for matching alterations to clinical trials (select all that apply)? *

Check all that apply.

- [ ] Clinicaltrials.gov
- [ ] WHO International Clinical Trials Registry
- [ ] Local or regional clinical trials databases
- [ ] Internal institutional database
- [ ] Personal network (i.e. no database but the treating physician knows that there are certain studies going on)
- [ ] I'm not sure
- [ ] Clinical trials are not included in the report

Other: [ ] ______________________

40. 34F. Which alteration-specific, targeted therapy types are reported (select all that apply)? *

Check all that apply.

- [ ] Approved therapies (on-label)
- [ ] Approved therapies (off-label)
- [ ] Therapies associated with lack of benefit i.e. resistance or safety impact
- [ ] Combination therapies i.e. pathway matches
- [ ] Clinical trials (investigational therapies)
- [ ] Newly emerging therapies from clinical trials outcome data
- [ ] Newly emerging therapies from preclinical study data
- [ ] I'm not sure

Other: [ ] ______________________

41. 34G. What tactics or methods, if any, are used to validate the results of external molecular testing (select all that apply)? *

Check all that apply.

- [ ] N/A: I don't validate results of external molecular testing
- [ ] Validate reported alterations by running a separate in-house molecular test as a confirmatory comparison
- [ ] Validate reported clinical trials by searching clinical trials databases (e.g. clinicaltrials.gov, WHO, etc.)
- [ ] Google or Pubmed searches
- [ ] Public database searches (e.g. COSMIC, MyCancerGenome, etc.)

Other: [ ] ______________________

42. 34H. In the interpretation of molecular data for the MTB, generally, which evidences are used predominantly for clinical vs. research applications (select all that apply)?

Check all that apply.

	Clinical	Research
Variant classification(s)	☐	☐
Origin (somatic vs. germline)	☐	☐
VAF (variant allele frequency)	☐	☐
Tumor purity / tumor cellularity	☐	☐
Family history / Hereditary disease association(s)	☐	☐
Variant databases (e.g. ClinVar, COSMIC, or Internal)	☐	☐
Clinical practice guidelines	☐	☐
Clinical trials databases	☐	☐
Published clinical studies	☐	☐
Radiology images	☐	☐
Pathology images	☐	☐
Protein biomarker data	☐	☐
RWE (real world evidence)	☐	☐
Multi-omics	☐	☐

MTB Tools & Performance

43. 35. MTB Preparation: What tools are used specifically for molecular data interpretation (select all that apply)? *

Check all that apply.

- [ ] Molecular data interpretation is only performed externally by a 3rd party provider
- [ ] Commercial variant interpretation software
- [ ] "Home brew" variant interpretation software, scripts, or algorithms
- [ ] Google searches
- [ ] Publication searches (e.g. Pubmed)
- [ ] Database / Knowledgebase searches (clinicaltrials.gov, COSMIC, OnkoKB, JaxCKB, MyCancerGenome, etc...)
- [ ] EMR (Electronic Medical Record)
- [ ] I'm not sure

Other: [ ] ____________________

44. 36. MTB Preparation: Is there a dedicated platform on which molecular data are brought together with other types of data (e.g. clinical data) in preparation for the MTB discussion? *

Mark only one oval.

- ( ) Yes
- ( ) No
- ( ) I'm not sure
- ( ) Other: ____________________

45. 37. MTB Presentation: What tools are used for presentation of data in the MTB meeting (select all that apply)? *

Check all that apply.

- [ ] Projected documents, spreadsheets, slides, websites
- [ ] Multi-Disciplinary Team / Tumor Board (MDT/TB)-specific software
- [ ] Variant interpretation software (commercial or home brew)
- [ ] Hard copy materials / hand-outs

Other: [ ] ____________________

46. 38. MTB Decision Documentation: How are MTB decisions and actions documented (select all that apply)?

Check all that apply.

	MTB recommendations	Administered therapy	Patient outcomes
Internal knowledge base entry (inc. Excel)	☐	☐	☐
EMR entry	☐	☐	☐
Lab Information Systems (LIMS / LIS) entry	☐	☐	☐
Manual report creation (e.g. microsoft word)	☐	☐	☐
Entry into MDT/TB-specific software	☐	☐	☐
Entry into variant interpretation software (commercial or home brew)	☐	☐	☐
Paper documentation	☐	☐	☐

47. 39. MTB Decision Access: To what degree are MTB documented decisions accessible to participants post the meeting (select all that apply)? *

Check all that apply.

☐ All meeting participants (including external participants) can access prior decisions
☐ All meeting participants (excluding external participants) can access prior decisions
☐ Only a small subset of meeting participants can access prior decisions
☐ N/A- Prior decisions are not routinely accessed
☐ I'm not sure
Other: ☐ ____________________

48. 40. How are MTB decisions communicated to clinicians and / or patients (select all that apply)? *

Check all that apply.

- [ ] Formal report
- [ ] Face-to-face meeting
- [ ] Phone call, email, or letter
- [ ] Separate genetic counseling
- [ ] Patient portal (via web or mobile app)
- [ ] I'm not sure

Other: [ ] ______________________

49. 41. What types of system or technology solution(s) are in place to capture and track patient status post MTB decision (select all that apply)? *

Check all that apply.

- [ ] Custom "in-house" solution(s) (e.g. internal knowledge base)
- [ ] Commercial software solution(s)
- [ ] Excel-type solution(s)
- [ ] EMR
- [ ] I'm not sure

Other: [ ] ______________________

50. 42. What metrics (KPIs) are used to track MTB use and impact (select all that apply)? *

Check all that apply.

- [ ] # of patients who received molecular testing
- [ ] # of patients who harbored at least 1 actionable alteration
- [ ] # of patients with actionable alterations who were matched to approved therapies
- [ ] # of patients with actionable alterations who were matched to off-label therapies
- [ ] # of patients with actionable alterations who were matched to clinical trials
- [ ] Response rates of therapies recommended in the MTB (e.g. overall, objective, partial response rates, duration, time to progression)
- [ ] Outcomes (e.g. PFS, ES, OS)
- [ ] Time from initial diagnosis (or relapse) to MTB therapy recommendation
- [ ] Adherence to MTB-recommended therapy recommendations
- [ ] Not currently tracking these types of metrics
- [ ] I'm not sure

Other: [ ] ______________________

51. 43. What is the typical turnaround time for the listed activities in the MTB journey (from molecular testing to start of therapy)? *

Check all that apply.

	1 week	2 weeks	3 weeks	4 weeks	above 4 weeks	I'm not sure
Time to completion of molecular analysis	☐	☐	☐	☐	☐	☐
Time to MTB discussion	☐	☐	☐	☐	☐	☐
Time to start of targeted therapy	☐	☐	☐	☐	☐	☐

52. 44. What do you deem to be the rate-limiting step(s) in getting to a timely MTB recommendation (select all that apply)? *

Check all that apply.

- ☐ Identification of appropriate patient candidates for molecular testing (i.e. patient accrual)
- ☐ Sample collection
- ☐ Laboratory testing (NGS and other tests)
- ☐ Turnaround time from third party laboratory
- ☐ Variant interpretation
- ☐ Assembling the MTB
- ☐ Arriving at an MTB decision
- ☐ Limited therapeutic options based on patient's insurance plan
- ☐ Documenting and communicating the MTB decision
- ☐ I'm not sure

Other: ☐ ____________________

53. 45. Of therapy recommendations made in the MTB, approximately what percentage are implemented? *

Mark only one oval.

- ( ) Less than 25%
- ( ) 25%
- ( ) 50%
- ( ) 75%
- ( ) 100%
- ( ) I'm not sure

54. 46. What are reasons for not implementing MTB therapy recommendations (select all that apply)? *

Check all that apply.

- [ ] N/A
- [ ] Medical judgment
- [ ] Insurance provider has declined due to "off label" or emerging / investigational nature of the therapy recommendation
- [ ] Patient has declined due to financial reasons
- [ ] Patient has declined due to cultural beliefs
- [ ] Patient has declined due to fear or lack of understanding of their therapy recommendation
- [ ] Patient has died
- [ ] I'm not sure

Other: [ ] ______________________

55. 47. In what way are MTB decisions enabling access and/or reimbursement for targeted therapies (select all that apply)? *

Check all that apply.

- [ ] MTB decisions are linked to reimbursement (has codes for reimbursement)
- [ ] MTB decisions are not linked to reimbursement
- [ ] Other multi-disciplinary team meetings (separate from the MTB) in the same institution are linked to reimbursement
- [ ] MTB decisions enable clinical trials enrollment
- [ ] MTB decisions in this institution influence or drive network consensus for access and/or reimbursement
- [ ] I'm not sure

Other: [ ] ______________________

56. 48. In what capacity is RWE (real world evidence) from clinical practice being used or soon will be used to support MTB decisions (select all that apply)? *

Check all that apply.

- [ ] Patient similarity analytics
- [ ] Tracking outcomes
- [ ] Clinical trial control groups (e.g. synthetic control arm)
- [ ] Providing supporting evidence for insurance coverage / preauthorizations
- [ ] RWE is not being used
- [ ] I'm not sure

Other: [ ] ____________________

Skip to question 57

Optional Final Thoughts

In your responses, please do not include any confidential or proprietary information about you, your institution, or patients.

57. 49. If theoretically (or in reality) you have been put in charge of personnel and resources for your institution's precision oncology (molecular) program, what would you remove, keep the same, or add in order to improve it? Which challenges would you aim to overcome?

58. 50. Please take this opportunity to provide any additional comments regarding MTBs that may not have been covered by the previous questions.

59. 51. If you are open to being contacted for a short follow-up discussion on this survey (e.g. to expand on specific topics or seek clarification on specific questions), please provide your email address. This action is voluntary. By providing this email address, you are agreeing that Roche may contact you for a brief follow-up discussion based on your responses to this survey. Roche will not use your email address for any other purpose without your express consent.

This content is neither created nor endorsed by Google.

Google Forms
